# Willie

**Published:** 1886-11-27

**Authors:** 


### WILLIE: A TRUE STORY.

By a Cottage Hospital Nurse.

How it all happened I can scarcely tell you. Do any of us know *really* how any of those accidents which leave us desolate and in life-weariness really happen?

He was an only son, and the idol of his mother. The early summer sunshine was smiling gladness into his sunny eyes and playing round his bright young head, when, in the light-heartedness of youthful buoyancy, her darling left her. In the old farm-garden the quaint, old-fashioned flowers, which had grown with him from babyhood and frolicked with him, nodded their farewells; the white roses which climbed about his bedroom window seemed to love him, since they thrust their budding fragrance through the open casement. Even the sentinel white lilies relaxed for him their wonted stateliness (for they knew he loved them) and waved him their adieux—for the last time, if they had but known it.

It was early morning, but not too early for a mother's love. His mother was up and stirring. With her own hands she had prepared his breakfast, and when his horse was ready she stood within the rose-girt doorway and watched him, proudly, fondly, while he vaulted into the saddle. That horse was a favourite with both, and while the boy caressed the flowing mane, the mother reached down a pendent rosebud and placed it in his button-hole. Then followed happy badinage, and when he stooped to kiss her, his lips were smiling. It was a fresh and joyous laugh which rang out on the morning breeze, to die only with the last hoof-echo.

He was a noble-hearted lad, and one who might atone amply for many a drawback in domestic comfort. For even in that sweet farmstead, where the roses flowered so profusely and the doves cooed so peacefully, there were jarring elements, and clouds, and thorns. The farmer was *his own worst enemy.* He had a good heart and many admirable traits, but drink enslaved him, and in a thousand ways his wife and family were sufferers therefrom. But Willie had no vices. His father's virtues were abundantly his own, defaced by no suspicion of his father's weakness. It was little wonder that his mother worshipped him.

It was fair-day—the great horse fair for which Horncastle is famous, and it was towards Horncastle that Willie journeyed. Was it in kindliness or irony that the sunshine was so bright and business ran so smoothly? In the matter of horseflesh he was something of a critic, and he enjoyed most keenly the bustle and activity which were rampant everywhere. There is a certain picturesqueness, in spite of squalor and tawdry finery, in the flashy costume even of English gipsies; even in the shameless lying and coarse chaffering of our horse-dealers there is an undercurrent of racy humour. There were, on this day, the accustomed bickerings, the flagrant cheatings and grossly-planned deceptions, too patent to deceive any save the most credulous Moses Primrose or chivalrous Don Quixote; but, to some extent, the genial sunshine threw a glamour over questionable practices. On such days Jews grow merciful.

To a horse-lover the animation of the scene was eminently fascinating. The variety was infinite, but for its full relish the presence of a horse-lover was necessary. There were serviceable draught-horses, strong, patient, willing, fleet racers, useful cobs, broken-down circus-coursers, still smacking of the stage, and as full of airs and graces as a superannuated belle, and one magnificent mare, black as night, agile, graceful, gentle (?), created first and foremost to take captive a young man's fancy. But the owner recognised the value of his animal, and priced it in accordance. Perhaps Willie was a cunning purchaser; perhaps the dealer was a novice, with a heart otherwhere than in his waistcoat pocket; perhaps he had a grain of conscience, and was willing thus to tacitly acknowledge the drawbacks of the favourite; perhaps he was drunk—in short, there is no end to the possible perhapses. Be that as it may, he abated his demands, and the mare changed masters. His new purchase appeared to Willie a fitting subject for congratulation, one of those bargains of red-letter day occurrence. He gloated over and gloried in it.

Then a friend met him—a neighbouring farmer.

"Two horses and one rider?" was his query. "I am in luck's way, Willie, for I came on foot. Let me ride the mare for you."

"My *own* mare, and welcome!" was the answer, "but my Cleopatra will out-distance you."

"No!—for a 'quid.' Shake hands on it."

Willie declined the bet, but he kept his opinion, and in the highest spirits they journeyed homewards.

There had been silence for some minutes, and Cleopatra had fallen back.

"We shall win you yet!" laughed Teesdale; "old Janet is bad to beat!"

But, meeting with no answer, he turned his head, to see with terror a plunging brute, kicking, furious, and riderless, for Willie lay upon the dusty highway bleeding and unconscious. How it happened or what followed who shall tell us? Not the dear, cold lips which shall speak to us no more; not the agonized and horror-stricken friend. And yet, somehow and by some means, he was carried to the bare, uncomfortable inn, and laid upon the hard floor-mattress. The news was wild-fire in that quiet village, and long before the doctor could be summoned men and women of many grades had flocked to the abode. Curious faces gathered round him, and curious tongues questioned and gossiped and told of other accidents. Women on tip-toe stole to him, and pitied and drew back, and looked again while again they pitied.

Many remedies were brought forward, and the tales repeated of wondrous cures. But Willie was unconscious of their idle chatter, for under his bandaged forehead a great wound was lying hidden. So with lamentations and morbid curiosity they made night hideous, to give place only, as the fever grew upon him, to strong men who held him down by force of muscle, and later, to his parents—his bewildered father and despairing mother—that night was one of those which render brown locks grey and young men old.

The village doctor was unequal to the fractured skull with all its delicate intricacies, and called in one of our more experienced staff. The boy was restless and consumed with fever, and in the midst of bustle and disorder. The village "public" was no place for an operation of so serious a nature, and removal was decided on—removal to our Cottage Hospital. That same evening I went for him, nor shall I soon forget the thronged inn, the eager sea of faces and dominant confusion. I shall remember, too, with all its details, that most anxious drive—the father on the box, and inside, their friend, my patient, and myself, then the operation and the long night-vigil. It was while I listened to each movement, and alternated betwixt hope and fear, that I learned to love him.

All that might be done we attempted for him. Poor lad! he was very patient, sometimes tossing feverishly, but for the most part in perfect quietude. How far he was really conscious it is hard to say. Once the application of some lotion wrung from him "It is very cold." On the second day he asked me, "Was it nearly dinner time?" and to my great happiness swallowed some beef-tea. The two other remarks he made, though trivial, were quite rational. But he never knew his mother, nor seemed conscious of his whereabouts. He would raise his hand to his head pitifully, and by that sign only betrayed that he was sensible of pain.

On the third morning after his admission he was worse; at nine that same sad evening he was at rest for ever. It brings the tears now when I think about it. I sat beside him in the sweet June twilight, applying ice and most intently watching. The mother came and the pity of it doubled, for her sorrow was heartrending. Her husband was all tenderness, and strove to calm her, but it was a cruel sight. If only her dear loved one could have opened his dim eyes and breathed her name, it would have been comfort to her. But no!

The end was very quiet. Of him it might be truly spoken—"His end was peace!" But the agony which followed it were vain to speak of. In losing him she felt that she was losing all, for it was her son rather than her husband on whom she leaned; and the father's grief was scarcely less, for he knew that his right hand had been taken from him.

> ". . . the good die first,
> And they whose hearts are dry as summer dust
> Burn to the socket."

He was so young—only seventeen—and his life had been so stainless! It was hard that he should perish thus with the sunshine on him, and the flowers which he loved all bursting into gladness, and the birds all breaking into song. It is so hard to be shut out in the darkness, life in ashes, and every cabined possibility no longer possible.

And I know that it was best, better far than the settled imbecility or gusts of furious passion which might have wrecked his future. It was best for him; best, too, it may be, for the friends who loved him; for, as sometimes death is mightier than life, so sometimes a cherished memory is more potent than the living presence. The shock was sobering, but not without its salutary influence. Willie's father is a better man—more considerate, temperate, and steady. In him "the white flower of one blameless life" has brought forth fruit.

In a pleasant Fenland churchyard, well known to one of England's greatest living novelists, Willie sleeps. There bush-grasses wave above him and autumn flowers breathe benedictions. And on the marble cross beneath his name and the dates which tell his earthly and his heavenly birthdays, are written words of promise: "Blessed are the pure in heart, for they shall see God!"

